# Regulation of Macrophage Polarization by miR-449a/Cripto-1-PI3K/AKT/NF-*κ*B Signaling Pathway in Allogeneic Transfusion Mice

**DOI:** 10.1155/2023/1277258

**Published:** 2023-01-06

**Authors:** Huan Wang, Na Yao, Man-di Wu, Ke Yue, Yu Bai, Lai-Wei You, Tong Liu, Fang Xu, Jian-Rong Guo

**Affiliations:** ^1^School of Basic Medical Sciences, Ningxia Medical University, Ningxia 750004, China; ^2^Postgraduate Training Base in Shanghai Gongli Hospital, Ningxia Medical University, Shanghai 200135, China; ^3^School of Medicine, Shanghai University, Shanghai 200444, China; ^4^Department of Anesthesiology, Shanghai Gongli Hospital, Naval Military Medical University, Shanghai 200135, China

## Abstract

In this study, the expression of Cripto-1 and the role of macrophage polarization in immune response after allogeneic transfusion were analyzed by constructing a mouse model of allogeneic transfusion. In order to analyze the effects of miR-449a on the PI3K/AKT/NF-*κ*B signaling pathway and the expression of downstream related regulatory factors under normal and abnormal conditions, we adopt in vitro and in vivo experiments separately. The molecular mechanism of PI3K/AKT/NF-*κ*B signaling pathway was analyzed by blocking or activating gene expression and western blotting. Experiment in vitro has confirmed that inhibition of miR-449a increased the protein expression of Cripto-1. In vivo experiments confirmed that allogeneic transfusion reduced the expression of Cripto-1, which further inhibited NF-*κ*B signaling pathway through AKT/PI3K phosphorylation, regulated macrophage polarization, inhibited M1 polarization of macrophages, promoted M2 polarization, and thus affected immune response of the body.

## 1. Introduction

Blood transfusion is an important clinical treatment and an irreplaceable therapeutic measure to save patients' lives [1, 2]. The potential risks of blood transfusion have also attracted much attention. For example, transfusion-related immunosuppression has become an important factor hindering the recovery and outcome of postoperative patients [2–5]. Transfusion of allogeneic blood leads to immunosuppression, and macrophages, as cells that perform innate immune function, have a close relationship with the immune function of the body. Allogeneic transfusion induces immune dysfunction in blood recipients by nonspecific and specific immunosuppression. The mechanism is generally believed that the inhibition of nonspecific immunity includes the inhibition of neutrophils, macrophages, natural killer cells, cytokines, and complement system [6, 7]. Macrophages are one of the important components of the innate immune system. Single allogeneic blood transfusion can induce the inhibition of mononuclear macrophage immune function in mice [8, 9]. Studies have shown that under different microenvironments, different stimuli can induce different polarization of macrophages, and different phenotypes play different pro-inflammatory or anti-inflammatory effects after activation [10, 11].

The polarization characteristics of macrophages are mainly caused by M1 type showing strong proinflammatory effect, while M2 type dominates anti-inflammatory response. It is currently believed that M1 and M2 are the two extremes of macrophage polarization, and macrophages are in dynamic change in most pathological states. Under normal circumstances, M1 and M2 are in a state of dynamic equilibrium, avoiding excessive polarization to maintain the body's steady state [12]. The polarization of macrophages is a complex process of multifactor interaction, which is regulated by a variety of intracellular signaling molecules and related pathways. Therefore, it is important to fully clarify the polarization subtypes of macrophages and their polarization mechanisms for clarifying the immunosuppression after allogeneic transfusion.

The polarization of macrophages is regulated by a variety of microbial signals and cytokines. Cripto-1 has been confirmed to play a regulatory role in the secretion and phagocytosis of macrophages during immune response. At the same time, macrophages themselves have high plasticity and heterogeneity, so we have guessed that Cripto-1 has a regulatory role in the polarization of macrophages and can affect the immune expression of patients. Our previous study found that miR-449a, miR-222, and miR-31 were correlated with Cripto-1mRNA 3′UTR, among which miR-449a was one of the miRNA associated with immune expression in the body [13, 14]. By analyzing the expressions of miR-449a and Cripto-1 after allogeneic transfusion, we further clarified the regulatory role of miR-449a on Cripto-1 protein, which is of great importance for reducing immunosuppression after allogeneic transfusion to find a definite target.

Cripto-1 protein is involved in gene transcription and protein synthesis through PI3K/AKT signaling pathway [15]. PI3K/AKT pathway is an indispensable influence of intracellular signal cascade on cell cycle, which participates in cell proliferation, quiescence, etc. When PI3K is activated, it triggers many reactions and, finally, phosphorylates and activates AKT [16]. PI3K is divided into 3 categories, among which, PI3K class I (PI3KI) is mainly involved in immune response and inflammatory response. Phosphorylation of PI3K results in PIP3 (phosphatidylinositol 3-phosphate), which binds to downstream AKT as a second messenger to promote translocation and phosphorylation. Phosphorylation of AKT1 regulates micRNA to further activate the downstream factor NF-*κ*B and inhibit the negative regulator of cytokine signal transduction 1 (SOCS-1), promoting the expression of M1 gene in macrophages [17]. NF-*κ*B gene is involved in different processes, especially regulation of immune system response [18]. Activation of the NF-*κ*B signaling pathway leads to further release of inflammatory cytokines such as TNF-*α* and IL-6 [19, 20]. Studies have shown that stimulation of macrophages with purified cripto-1 significantly increased the level of phosphorylated nuclear factor-*κ*B inhibitor kinase (P-IKK) in macrophages. Cripto-1 activates the NF-*κ*B signaling pathway in macrophages [21]. After treatment with PDTC, an inhibitor of NF-*κ*B signaling pathway, Cripto-1 significantly inhibited the secretion and phagocytic functions of macrophages [14]. On the other hand, AKT2 can make macrophages have M2-type phenotype by regulating the expression of Arg-1 [22].

**Figure 1:**
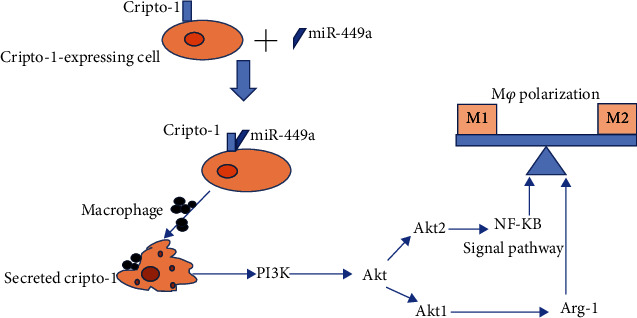
Schematic diagram of Cripto-1 involving in PI3K/AKT/NF-*κ*B signaling pathway to affect the polarization of macrophages.

Based on the above close association between miR-449a/Cripto-1, PI3K/AKT/NF-*κ*B signaling pathway, and polarization of macrophages, we propose a hypothesis. Under normal conditions, Cripto-1 is not expressed and does not induce cascade activation of PI3K/AKT/NF-*κ*B signaling pathway. Macrophage polarization level is maintained in equilibrium between M1 and M2, and normal immune response is maintained in the body. However, after allogeneic transfusion, the organism's microenvironment changes. miR-449a/Cripto-1 activates AKT through PI3K phosphorylation, which further activates lower NF-*κ*B and regulates macrophage polarization through NF-*κ*B signaling pathway, thereby affecting immune responses (Figure 1). Through the above studies, the role of Cripto-1 in promoting immunosuppression after allogeneic transfusion and its molecular mechanism can be preliminary clarified, and it is expected to provide a new possible target for clinical prevention and treatment of immunosuppression after allogeneic transfusion.

## 2. Materials and Methods

Materials: DMEM (Hyclone). Fetal bovine serum (Gibco). PBS, 0.25% trypsin (Hyclone). RIPA lysate (Beijing Solebo). SYBR green PCR kit (Thermo); Trizol (invitrogen). BCA Protein Quantitation Kit (Thermo). Tris-HCl, pH = 8.8 electrophoretic buffer solution (Beijing Solebo). CO2 constant temperature incubator (Thermo). Real-time detector (ABI) (ABI-7300). Flow cytometry (Accuri C6, BD). Refrigerated centrifuge (TG-16M) (Shanghai Luxiangyi Centrifuge Instrument Co., Ltd.). Consumables for cell culture (Corning). C57BL/6J SPF level mice (4 week, 13-16 g) were purchased form Weitong Lihua Laboratory Animal Technology Co., Ltd. All animals were maintained on an ad libitum diet of laboratory chow and water and approved by the × × University of Animal Care Committee.

### 2.1. Methods

#### 2.1.1. Construction of Cripto-1 Overexpressed and Knockout Mice

Construction of Cripto-1 knockout mouse: oligo DNA was designed in the first exon region of cripto-1 CDS region on http://crispr.mit.edu/ website, and the plasmid vector containing Cripto-1 was constructed. Then, the vector DNA was retrotranscribed into mRNA and microinjected into the fertilized eggs of mice for in vitro incubation. When the fertilized eggs were cultured to blastocysts in vitro, they were transplanted to pseudopregnant mice. After the F0 generation gave birth to the F1 generation, the genome was extracted to detect gene mutation types, and the Cripto-1 gene knockout homozygous mice were obtained through hybridization screening. Construction of Cripto-1 overexpressed mice: Cripto-1 associated virus (synthesized by Shanghai Jima Company) was transfected into C57BL/6J mice by tail vein injection.

#### 2.1.2. Allogeneic Transfusion and Grouping

Allogeneic transfusion in Cripto-1 overexpressed mice and knockout mice was performed. Mice were intraperitoneally injected with 2 mg/kg lipopolysaccharide. Two hours later, abdominal anesthesia and femoral artery cannula were performed. Within 5-10 min, 10% of the total body blood volume (0.1 mL) was removed and then injected equal volume of plasma at an infusion rate ≤ 0.1 mL/h. Mice in control group were intraperitoneally injected with normal saline [23]. Groups are (1) control, (2) si-Cripto-1 (Cripto-1 knockout), (3) si-NC (knock out control group), (4) p-Cripto-1 (Cripto-1 overexpression), (5) p-NC (overexpression control group), (6) allogeneic transfusion (AT), (7) AT+ si-Cripto-1, (8) AT+si-NC, (9) AT+p-Cripto-1, and (10) AT+p-NC.

#### 2.1.3. Cripto-1 mRNA in Animal Blood Was Detected by RT-PCR

Animal blood was collected, and Trizol cell lysate of 3 times volume was added. The samples were prepared a homogenate by homogenizer on ice for 30 s. The samples were centrifuged for 15 min at 12000 r/min. Total RNA was extracted, and 20 *μ*L DEPC was added to dissolve RNA. Primer design and cripto-1 primer sequence: primer F 5′ TCTGAATGGAGGGACTTG 3′; primer R 5′ CACAGGGAACACTTCTTG 3′. RNA reverse transcription reaction, reaction procedure: 42°C, 60 min; 70°C, 5 min; and store at -20°C. Real-time PCR amplification, reaction conditions: 95°C, 10 min (95°C, 15 s; 55°C, 45 s) × 40. The mRNA relative expression of target gene was calculated by 2-*ΔΔ*Ct method.

#### 2.1.4. Cripto-1 Expression in Animal Blood Was Detected by Western Blot

Blood was collected from the abdominal aorta, centrifuged, and the precipitate was collected. Precipitate (10 *μ*L) and SDS-PAGE protein loading buffer (4 *μ*L) were mixed. The protein was fully denaturated by heating at 100°C for 10 min. The supernate was obtained by centrifuging at 5000 × g for 1 min. The supernate was added to the sample well and changed the sample volume to ensure the same amount of protein in each lane. The proteins were separated by SDS-polyacrylamide gel electrophoresis. After the protein being blocked, the primary antibodies (Cripto-1 and *β*-actin) were incubated at 1 ∶ 1000 dilution for 2 h at room temperature. After TBST washing, HRP-labeled secondary antibodies were incubated at room temperature for 1 h. Cripto-1 expression was scanned with Image J software and quantified with *β*-actin gray value.

#### 2.1.5. Markers of Macrophage Polarization in Animal Blood Were Detected by ELISA

Markers of macrophage polarization in animal blood were detected by ELISA, M1 (iNOS, TNF-*α*, IL-1*β*, and IL-6) and M2 (Arg-1 and IL-10). The specific operation refers to ELISA kit. Set blank hole and sample hole to be tested, respectively. Add sample 10 *μ*L to the bottom of enzyme plate well, shake gently, and mix well. The samples were incubated at 37°C for 30 min. The liquid was discarded and washed with PBS for 5 times. Add HRP-conjugate reagent 50 *μ*L to each well and incubated at 37°C for 30 min. Chromogenic agent A 50 *μ*L and chromogenic agent B 50 *μ*L were added and mix gently and keep away from light for 15 min at 37°C. OD value of the samples was detected at 450 nm.

#### 2.1.6. Detection of Mitochondrial Membrane Potential in Macrophages

Mice peritoneal fluid macrophages were collected, and the cell concentration was adjusted to 2 × 10^6^/mL with precooling medium. The cell suspension was cultured in a 24-well culture plate and incubated at 37°C with 5% CO_2_ for 2-4 h. The culture supernatant was discarded, and the cells were gently washed with RPMI1640 for 1-2 times. The nonadherent lymphocytes and other cells were fully discarded, and the adherent cells were obtained as monolayer macrophages [24]. The prepared cell suspension was taken out and centrifuged at 1500 rpm for 10 min. Cell precipitates were collected, and the cell concentration was adjusted to 1 × 10^6^ cells/mL. 0.5 mL JC-1 staining solution was added to each experimental group and mixed several times upside down. The cells were incubated at 37°C for 20 min and detected by BD flow cytometry.

#### 2.1.7. Activity of Mitochondrial Respiratory Chain Complex I, I, III, and IV Was Detected by ELISA

The contents of inflammatory factors in macrophages were determined by ELISA, and the concentrations of mitochondrial respiratory chain complex I, II, III, and IV in cell homogenates were calculated. All operations were performed according to the instructions of the mitochondrial respiratory chain complex I activity detection kit.

#### 2.1.8. Cell Transfection

The plasmids miR-449a and Cripto-1 were chemically synthesized by Shanghai Jima Company. The macrophages were planted in a 6-well culture plate and placed in a cell incubator for continuous culture. Transfection was performed when the cell density was about 50-60%. The corresponding oligoucleic acid was mixed into OPti-MEMI to obtain the transfection mixture. After 15 min of incubation, the medium in the six-well plate was replaced with the transfection mixture. The medium was placed in a 5% CO_2_ incubator at 37°C for further cultivation and was replaced with the complete medium after culture addition. The cell groups were divided into control, miR-449a-mimics, mimics-NC, miR-449a-inhibitor, inhibitor-NC, miR-449a-inhibitor+si-CR-1, and miR-449a-inhibitor+si-NC. Three weeks later, single cell clones were collected, cell supernatant was collected after amplification, and cytoplasmic proteins were extracted. Cripto-1 mRNA and Cripto-1 protein expressions were detected by RT-PCR and western blot.

#### 2.1.9. Markers of Macrophage Polarization In Vitro

Markers of macrophage polarization in animal blood were detected by ELISA, M1 (iNOS, TNF-*α*, IL-1*β*, and IL-6), and M2 (Arg-1 and IL-10). The specific operation refers to ELISA kit. Set blank hole and sample hole to be tested respectively. Add sample 10 *μ*L to the bottom of enzyme plate well, shake gently, and mix well. The samples were incubated at 37°C for 30 min. The liquid was discarded and washed with PBS for 5 times. Add HRP-conjugate reagent 50 *μ*L to each well, and incubate at 37°C for 30 min. Chromogenic agent A 50 *μ*L and chromogenic agent B 50 *μ*L were added and mix gently, keep away from light for 15 min at 37°C. OD value of the samples was detected at 450 nm.

#### 2.1.10. CD86 and CD206 Were Detected by FCM

Cells are digested with trypsin, and cell deposits are collected. The concentration of cells to be detected was adjusted to 10^6^ cells/mL, 200 *μ*L was taken, and centrifuged at 1000 rpm for 5 min. Precooled PBS was rinsed 1 mL twice, and the cells were suspended in 100 *μ*L PBS. 3 ~ 5 *μ*L CD86 and CD206 antibodies were added, mixed gently, and incubated at 4°C for 1 ~ 2 hours away from light. After washing with 400 *μ*L PBS, flow cytometry was used for detection.

#### 2.1.11. Expression of AKT/PI3K/NF-*κ*B Pathway-Related Proteins

Western blot was used to detect the expression of PPI3K and NF-*κ*B pathway-related proteins. Blood was collected from the abdominal aorta, centrifuged, and the precipitate was collected. Precipitate (10 *μ*L) and SDS-PAGE protein loading buffer (4 *μ*L) were mixed. The protein was fully denaturated by heating at 100°C for 10 min. The supernate was obtained by centrifuging at 5000 × g for 1 min. The supernate was added to the sample well and changed the sample volume to ensure the same amount of protein in each lane. The proteins were separated by SDS-polyacrylamide gel electrophoresis. After the protein being transferred and sealed, the primary antibodies (PPI3K, PAKT, NF*κ*B, and *β*-actin) were incubated at 1∶1000 dilution for 2 h at room temperature. After TBST washing, HRP-labeled secondary antibodies were incubated at room temperature for 1 h. PPI3K, PAKT, NF*κ*B expression were scanned with Image J software and quantified with *β*-actin gray value.

## 3. Results

### 3.1. Effects of Cripto-1 on Inflammatory Cytokine Secretion and Macrophage Polarization after Allogeneic Transfusion

#### 3.1.1. Results of PCR

**Figure 2:**
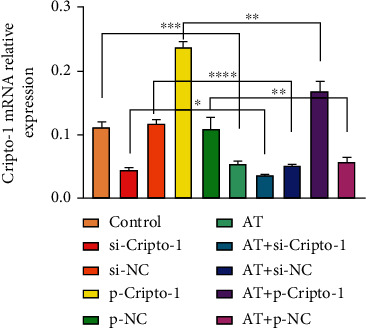
Cripto-1 mRNA relative expression in different groups. Statistical comparison between groups, ^∗^*P* < 0.05, ^∗∗^*P* < 0.01, ^∗∗∗^*P* < 0.001, and ^∗∗∗∗^*P* < 0.0001.

Compared with the control group, p-Cripto-1 and AT+p-Cripto-1 showed high expression of Cripto-1 mRNA, while si-Cripto-1 and AT+si-Cripto-1 showed low expression. It indicated that Cripto-1 overexpression and knockout mice were successfully constructed. But Cripto-1 mRNA relative expression was much lower in AT group, AT+si-Cripto-1, AT+si-NC, AT+p-Cripto-1, AT+p-NC group, and ^∗∗∗^*P* < 0.001. It indicated that allogeneic transfusion could decrease the expression of Cripto-1 mRNA, as shown in Figure 2.

#### 3.1.2. Cripto-1 Expression in Animal Blood

**Figure 3:**
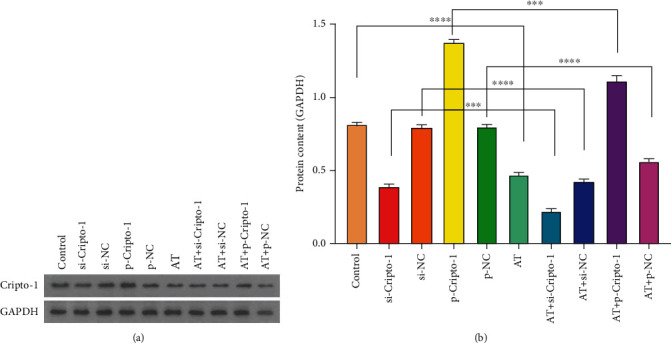
The expression levels of Cripto-1 protein in different groups. Compared with control, si-NC, si-Cripto-1, p-NC, p-Cripto-1, ^∗∗∗∗^*P* < 0.0001, and ^∗∗∗^*P* < 0.001.

The expression of proteins Cripto-1 was detected by western blot, as shown in Figure 3. Protein Cripto-1 in p-Cripto-1 and AT+p-Cripto-1 group all increased, but decreased in si-Cripto-1 and AT+si-Cripto-1. ^∗∗∗∗^*P* < 0.0001, and ^∗∗∗^*P* < 0.001. The trend of the expression levels of proteins Cripto-1 was p-Cripto-1 group>AT+p-Cripto-1 group>Control, si-NC, p-NC>AT>si-Cripto-1>AT+si-NC>AT+p-Cripto-1 group. It indicated that allogeneic transfusion could decrease the expression of Cripto-1 protein.

#### 3.1.3. Markers of Macrophage Polarization in Animal Blood

**Figure 4:**
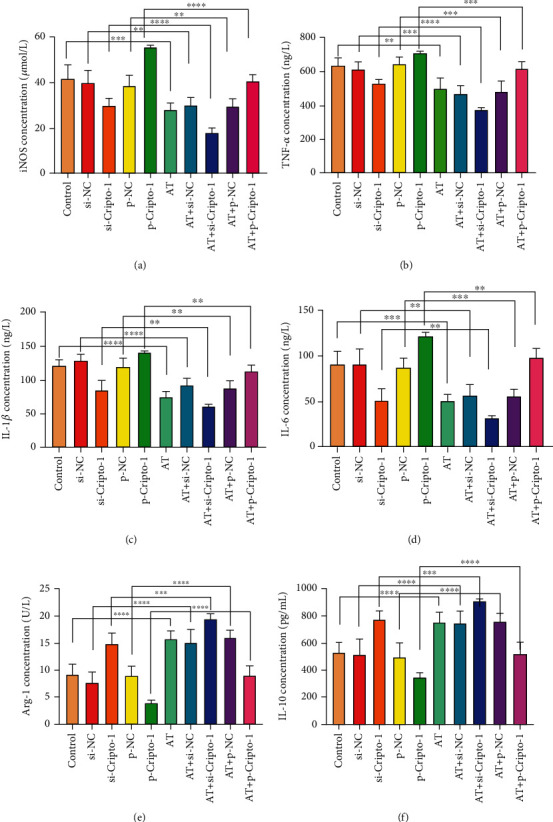
Inflammatory factor of macrophage polarization markers M1 and M2 in animal blood. (a) iNOS (umol/L); (b) TNF-*α* (ng/L); (c) IL-1*β*(ng/L); (d) IL-6(ng/L); (e) Arg-1 (U/L); (f) IL-10 (pg/mL). Compared with control, si-NC, si-Cripto-1, p-NC, p-Cripto-1, ^∗∗∗∗^*P* < 0.0001, ^∗∗∗^*P* < 0.001, and ^∗∗^*P* < 0.01.

The main inflammatory factors of M1 include iNOS, TNF-*α*, IL-1*β*, and IL-6. The contents of iNOS, TNF-*α*, IL-1*β*, and IL-6 decreased in AT group compared with control. AT group reduced the contents of iNOS, TNF-*α*, IL-1*β*, and IL-6, especially AT+si-Cripto-1 group. It suggested that Cripto-1 knockdown could reduce the contents of inflammatory factors in allogeneic transfused mice. Even in the p-Cripto-1 group and AT+p-Cripto-1 group, allogeneic transfusion could also reduce the expression of M1 markers, as shown in Figures 4(a)–4(d), ^∗∗∗∗^*P* < 0.0001, ^∗∗∗^*P* < 0.001, and ^∗∗^*P* < 0.01. M2 markers were mainly Arg-1 and IL-10, which were opposite to M1 markers. Allogeneic transfusion and Cripto-1 could increase contents of Arg-1 and IL-10, as shown in Figures 4(e) and 4(f), ^∗∗∗∗^*P* < 0.0001, ^∗∗∗^*P* < 0.001, and ^∗∗^*P* < 0.01. In a word, allogeneic transfusion increased M2 markers and decreased M1 markers. Overexpression of Cripto-1 increased M1 expression and decreased M2 marker expression, while knockdown of Cripto-1 was opposite.

#### 3.1.4. Mitochondrial Membrane Potential in Macrophages

**Figure 5:**
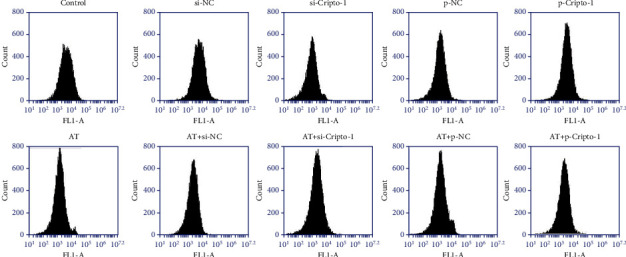
Detection of mitochondrial membrane potential in macrophages of different groups was detected by flow cytometry. Fluorescence channel selection FLI-A.

Trend of mitochondrial membrane potential of macrophages: p-Cripto-1 > Control = siNC = p-NC ≈ AT+p-Cripto-1 > si-Cripto-1 ≈ AT = AT+siNC = AT+p-NC > AT+si-Cripto-1. The results were shown in Figure 5. It indicated that increased allogeneic transfusion decreased MMP. Overexpression of Cripto-1 increased MMP and silencing of Cripto-1 decreased MMP.

#### 3.1.5. Activity of Mitochondrial Respiratory Chain Complex I, I, III, and IV

**Figure 6:**
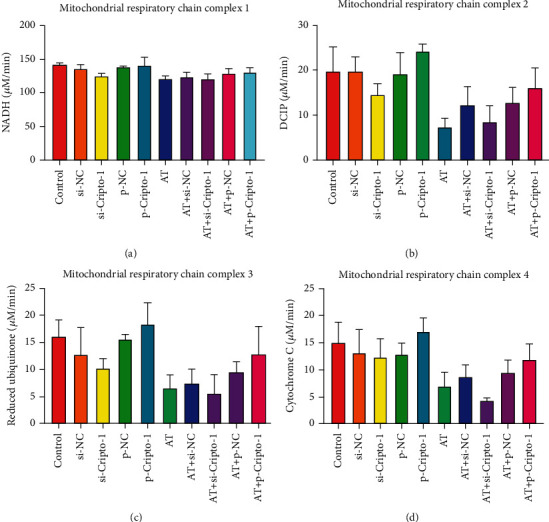
Activities of mitochondrial respiratory chain complex I, I, III ,and IV were detected by ELISA.

Trend of activity of mitochondrial respiratory chain complex I, I, III, and IV of macrophages: p-Cripto-1 > Control = siNC = p-NC ≈ AT+p-Cripto-1 > si-Cripto-1 ≈ AT = AT+siNC = AT+p-NC > AT+si-Cripto-1. The results were shown in Figure 6. It indicated that increased allogeneic transfusion decreased activity of complex I, I, III, and IV. Overexpression of Cripto-1 increased activity of complex I, I, III, and IV; and silencing of Cripto-1 decreased activity of complex I, I, III, and IV.

### 3.2. Cripto-1 mRNA and Protein Expression in Cells

**Figure 7:**
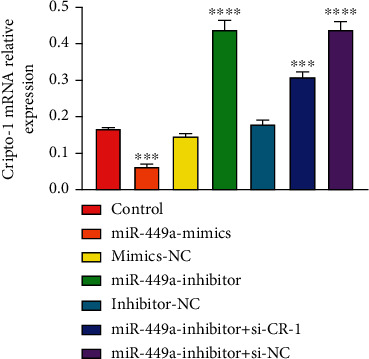
Cripto-1 mRNA relative expression in different groups.

**Figure 8:**
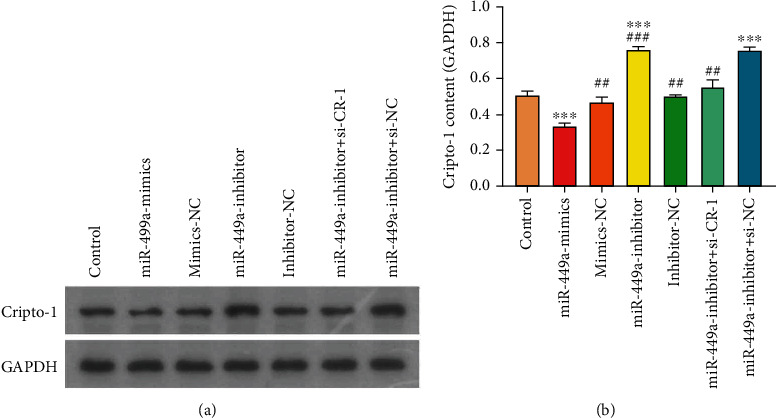
The expression levels of Cripto-1 protein in different groups. Compared with control, ^∗∗∗∗^*P* < 0.0001, and ^∗∗∗^*P* < 0.001.

The expression of Cripto-1 mRNA was detected by RT-PCR, as shown in Figure 7. Cripto-1 mRNA in miR-499a-inhibitor, miR-499a-inhibitor+si-CR-1, and miR-499a-inhibitor+si-NC group all increased, but decreased in miR-499a-mimics. ^∗∗∗∗^*P* < 0.0001, and ^∗∗∗^*P* < 0.001. It indicated that miR-499a negatively regulated Cripto-1. The trend of the expression levels of proteins Cripto-1 was detected by western blot, as shown in Figure 8, which showed the same trend as PCR results.

### 3.3. Markers of Macrophage Polarization In Vitro

**Figure 9:**
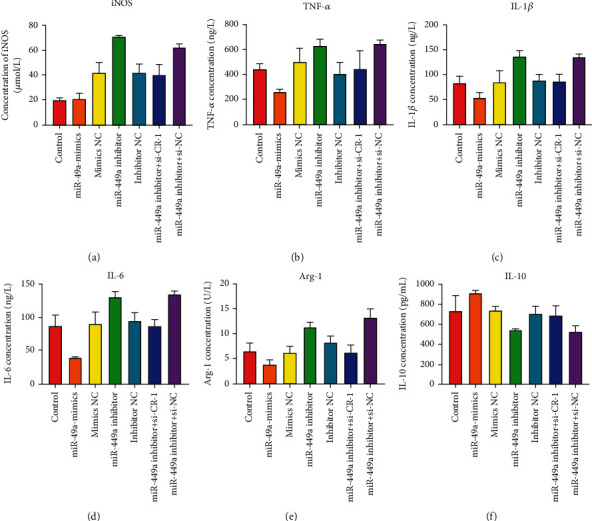
Inflammatory factor of macrophage polarization markers M1 and M2 in vitro. (a) iNOS (umol/L); (b) TNF-*α*(ng/L); (c) IL-1*β*(ng/L); (d) IL-6(ng/L); (e) Arg-1 (U/L); (f) IL-10 (pg/mL). Compared with control, si-NC, si-Cripto-1, p-NC, p-Cripto-1, ^∗∗∗∗^*P* < 0.0001, ^∗∗∗^*P* < 0.001, and ^∗∗^*P* < 0.01.

The contents of iNOS, TNF-*α*, IL-1*β*, and IL-6 increased in miR-499a-inhibitor and miR-499a-inhibitor+si-NC group (Figures 9(a)–9(d)). M2 markers were mainly Arg-1 and IL-10, which were opposite to M1 markers, as shown in Figures 9(e) and 9(f). Inhibition of miR-499a will promote macrophage M1. Overexpression of miR-499a promoted M2 polarization and inhibited M1 polarization.

### 3.4. M1 and M2 Markers CD86 and CD206 Were Detected by FCM

**Figure 10:**
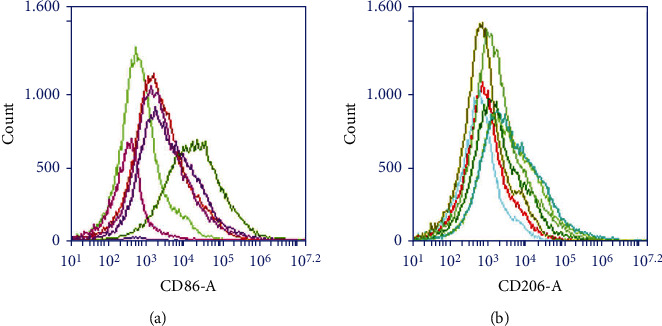
M1 and M2 markers CD86 and CD206 were detected by flow cytometry.

M1 (CD86): miR-449a inhibitor = miR-449a inhibitor + si-NC > inhibitor NC ≈ mimics NC ≈ miR-449a inhibitor + si-CR-1 ≈ Control >miR-449a mimics, as shown in Figure 10(a). M2 (CD206): miR-449a inhibitor = miR-449a inhibitor + si-NC < inhibitor NC ≈ mimics NC ≈ miR-449a inhibitor + si-CR-1 ≈ Control <miR-449a mimics, as shown in Figure 10(b). Overexpression of miR-449a promoted M2 polarization and inhibited M1 polarization.

### 3.5. Expression of AKT/PI3K/NF-*κ*B Pathway-Related Proteins

**Figure 11:**
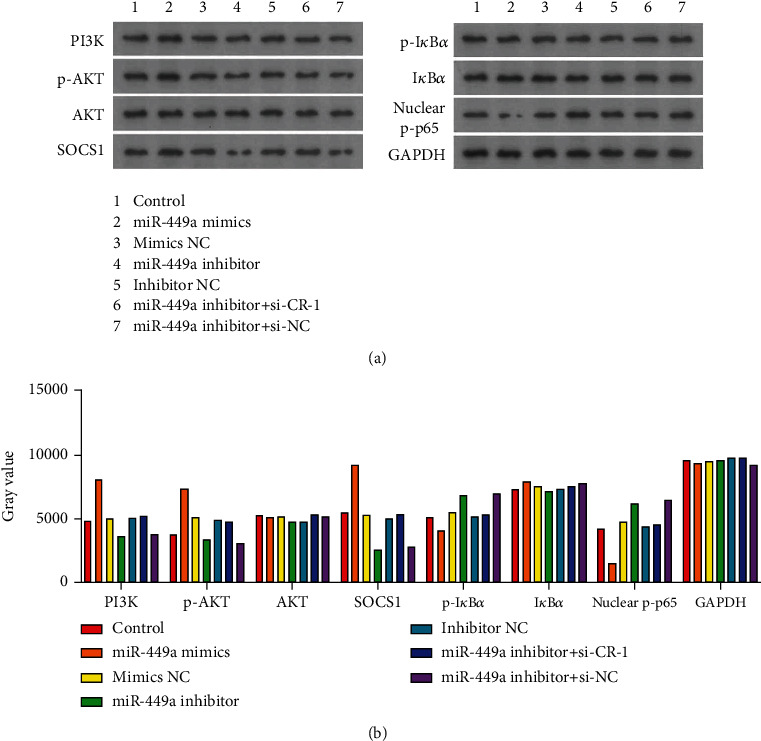
The expression levels of Cripto-1 protein in different groups. (a) Protein grey band. (b) Gray value.

The expression of PI3K, p-AKT, and SOCS1 was higher in miR-449a-mimics group compared with control group. While the expression of p-I*κ*B*α*和nuclear p-p65 was decreased, as shown in Figure 11. It indicated that miR-449 increased the expression of PI3K and p-AKT and SOCS1 and inhibited the expression of p-I*κ*B*α* and nuclear p-p65.

## 4. Discussion

Bioinformatics software was used to predict potential miRNA binding sites on Cripto-1 mRNA3′UTR, and double luciferase reporter gene was used to detect the binding activity of miRNA and Cripto-1 mRNA3′UTR. After verifying that miR-449a targets Cripto-1, overexpression of macrophages or siRNA interference with miR-449a, the expression of major inflammatory factors M1 and M2 of macrophage polarization markers in animal blood was detected. It was found that after red blood cell infusion, macrophages will show anti-inflammatory, namely, M2 polarization. The literature shows that overexpression of CR-1 can promote the anti-inflammatory factor IL-10, and proinflammatory factor IL-1 of macrophages *β*, TNF-*α*, IL-6 expression, and CR-1 also activates NF-*κ*B channel and increases expression of *κ*B.

The polarization of macrophages is regulated by a variety of microbial signals and cytokines, and the corresponding functional transformation occurs in the change of microenvironment. Cripto-1, as a multifunctional signaling molecule, exerts an extracellular immune effect through paracrine mode, such as regulating macrophage expression of proinflammatory cytokines TNF-*α* and IL-6, inducing chronic colitis [25]. Cripto-1 was low or not expressed in normal human tissue cells, but was abnormally high in some malignant tumor cells at the early stage of embryonic development. Cripto-1 has been confirmed to play a regulatory role in the secretion and phagocytosis of macrophages in the immune response. Meanwhile, macrophages themselves have a high degree of plasticity and heterogeneity, so Cripto-1 has a regulatory role in the polarization of macrophages and can affect the immune expression of patients. When pathogens invade, changes in the microenvironment lead to the transformation of macrophages into “M1” type, which produces a large number of inflammatory factors, kills the invading organisms, and activates adaptive immunity, resulting in strong proinflammatory ability [26]. At the same time, these inflammatory factors also induce apoptosis of macrophages or to “M2” type polarization, which has obvious anti-inflammatory effect. These two polarization types have different functions and even resist each other. The final polarization state of macrophages determines the final immune response of the body. After allogeneic blood transfusion, the microenvironment of the body changes, macrophages polarize to “M2” type, and there will be an obvious immunosuppression response, which can provide a new idea for the prevention and treatment of immunosuppression after allogeneic blood transfusion.

Cripto-1 functional domain plays an important role in signal transduction pathway and participates in signal transduction process as an auxiliary receptor [14, 21, 27]. Studies have shown that Cripto-1 protein has soluble secretory protein forms and membrane-anchored binding forms in a variety of cell lines and in vivo and can be used as a complex to receive multiple signal stimulation. It has been shown that overexpression of CR-1 can promote the expression of anti-inflammatory cytokines IL-10 and proinflammatory cytokines IL-1*β*, TNF-*α*, and IL-6 in macrophages, and Cripto-1 also activates NF-*κ*B pathway and increases the expression of I*κ*B. Cripto-1 promotes M1 polarization. Allogeneic transfusion should reduce Cripto-1 expression, consistent with the trend of macrophage polarization. The NF-*κ*B signaling pathway and AKT/PI3K signaling pathway cross-linked with allogeneic transfusion and may be involved in the polarization of macrophages, thereby affecting the immune response after allogeneic transfusion.

## 5. Conclusion

Inhibition of miR-449a increased the protein expression of cripto-1. Allogeneic transfusion reduced the expression of Cripto-1, which further inhibited NF-*κ*B signaling pathway through AKT/PI3K phosphorylation, regulated macrophage polarization, inhibited M1 polarization of macrophages, promoted M2 polarization, and thus affected immune response of the body.

## Data Availability

The data are free access to available upon request.
